# Exosome-Derived Non-Coding RNAs in the Tumor Microenvironment of Colorectal Cancer: Possible Functions, Mechanisms and Clinical Applications

**DOI:** 10.3389/fonc.2022.887532

**Published:** 2022-05-12

**Authors:** Xian Chen, Mengmeng Jia, Jing Ji, Zhiying Zhao, Yanjie Zhao

**Affiliations:** School of Public Health, Qingdao University, Qingdao, China

**Keywords:** colorectal cancer, exosome, ncRNAs, tumor microenvironment, tumor biomarker

## Abstract

Colorectal cancer (CRC) is the second leading cause of cancer death and the third most prevalent malignancy. Colorectal tumors exchange information with the surrounding environment and influence each other, which collectively constitutes the tumor microenvironment (TME) of CRC. Many studies have shown that exosome-derived non-coding RNAs (ncRNAs) play important roles in various pathophysiological processes by regulating the TME of CRC. This review summarizes recent findings on the fundamental roles of exosomal ncRNAs in angiogenesis, vascular permeability, tumor immunity, tumor metabolism and drug resistance. Certainly, the in-depth understanding of exosomal ncRNAs will provide comprehensive insights into the clinical application of these molecules against CRC.

## Introduction

CRC is the third most common malignancy (1). Although data from the American Cancer Society showed a gradual decline in mortality rates for CRC, this trend hides a rise in mortality among young adults (2). With the death rate for young people with CRC rises, a better understanding of molecular pathogenesis and the exploration of sensitive surveillance tools for early diagnosis and therapy are critical.

CRC is widely regarded as a heterogeneous disease, and its pathogenesis involves multiple genetic changes and multiple pathways (3). Tumor heterogeneity causes differences between and within CRC, which also increases the difficulty in the treatment of CRC (4, 5). TME contributes significantly to this heterogeneity because it is the site of tumor cell formation and growth (6, 7). TME is an intricate system, composed of primary cancer cells, associated stromal and immune cells, which considerably affects the behavior of CRC cells at the primary tumor site as well as in metastatic lesions (8). More evidence indicates that exosomes may impact carcinogenesis and development as crucial players in the communication between tumor cells and surrounding components in TME (9, 10). According to a growing amount of scientific, lots of ncRNAs existing in exosomes play a regulatory role in various pathophysiological activities of CRC (11, 12). Furthermore, ncRNAs in exosomes can influence the malignant progression of tumorigenesis by a variety of ways, making them a research hotspot in recent years (10, 13).

In this review, we discussed the various functions and mechanisms of exosomal ncRNAs: microRNA (miRNA), long non-coding RNA (lncRNA), circular RNA (circRNA) in TME, thereby elucidating the possibility of exosomal ncRNAs being applied clinically to treat CRC.

## 1 The TME of CRC

Cancer progression is dependent on the capacity of tumor cells to establish a supportive TME (14). Studies have demonstrated that the microenvironment is involved in causing a condition of growth arrest in the tumor (15). At the same time, tumors fight against the normal microenvironment to overcome the anti-tumor pressure (15). While tumors communicate closely with the surrounding microenvironment, the biochemical signals in the microenvironment impact cell growth and tumorigenesis (16, 17). The normal microenvironment inhibits cancer progression in a steady state. On the contrary, when this steady-state structure is out of control, the microenvironment itself will send out tumor-promoting signals, promoting cell malignant transformation (18, 19).

TME is an important factor leading to the heterogeneity and targeted therapy of CRC (6, 20). The composition of TME is influenced by both tumor features and patient state, which impacts disease progression, responsiveness to cancer therapy, and survival prognosis (21). A lot of research shows that TME plays a crucial role in tumor growth, metastasis, and drug resistance (22). Components of the TME in CRC include colorectal tumor cells, blood vessels, fibroblasts, immune and inflammatory cells, the extracellular matrix, as well as many signaling molecules and pathways that impact the angiogenic response (18, 23, 24).

As the most abundant cell type in TME, CAFs regulate many aspects of tumorigenesis (25, 26). Studies have shown that cancer-associated fibroblasts (CAFs) contribute to CRC progression through immunosuppression, extracellular matrix (ECM) remodeling and promotion of epithelial mesenchymal transformation (EMT) (27). Colorectal tumor cells affect the recruitment of CAF precursors and induce the differentiation of normal fibroblasts into CAFs, promote tumor growth and maintain its malignant propensity (24). Suetsugu et al. found that colon metastatic tumor cells can recruit CAFs to metastatic sites and contribute to tumor progression (28). In addition, the TME shows great diversity in different types of cancer (29). In terms of immune cells in the TME of colon cancer, high tumor-associated macrophages (TAMs) are associated with fewer liver metastases (30). However, another study showed that high TAM is associated with a higher clinical stage in the TME of esophageal cancer (31). While high TAM is associated with poorly differentiated histology and lymph node metastasis in cholangiocarcinoma (32). Ugai et al. found the immunological microenvironment were different between moderate and advanced CRC patients. Moreover, lymphocytic response patterns, macrophages, and regulatory T cells in the TME were associated with patients’ age. Thus, immune cell profiles by age of diagnosis may help to explain the growth and progression of CRC in young people (33).

Tumor and stromal cells located in the TME can secrete both various soluble molecules and vesicles, including exosomes (24). Exosomes have been explored as key factors mediating cell-to-cell communication between tumor cells and the microenvironment, which are involved in various signaling pathways regulated in the TME. Hence, exosomes in the TME could be a promising therapeutic target for CRC therapy (34).

## 2 Overview of Exosomes

### 2.1 Composition and Biogenesis of Exosomes

Exosomes were first described in the 1980s as membranous vesicles in reticulocytes (35). Initially, exosomes were considered cellular garbage, withal later studies have shown that exosomes can transfer genetic information to achieve cell-to-cell communication (36). The biogenesis of exosomes mainly involves three stages: first, the fusion of endocytic vesicles produces early endosomes (EEs), which encapsulate the cargo of endocytic cells that share certain biomolecules and membrane proteins; second, the late endosomes (LEs) are composed by the inward sprouting of the multivesicular body (MVB) membrane; finally, MVB can fuse with lysosomes or autophagosomes for degradation, which can also fuse with the plasma membrane to release the contained substances, namely exosomes (37, 38). They are encased in lipid bilayers and carry various biological molecules, such as RNA, DNA, proteins, glycans, and lipids (39). Many studies revealed that exosomes are rich in ncRNA (40). ncRNAs can bind to recipient target cells through exosome carriers to transmit information and change the gene expression and function of recipient cells, thus affecting cancer progression to a certain extent (34, 41).

### 2.2 Exosome-Derived ncRNAs: Participants in the TME of CRC

Exosome-derived ncRNAs are involved in driver mutations and epigenetic modifications that drive various pathophysiological processes in CRC (42). A large number of studies on exosomes have shown that exosomal ncRNAs communicate throughout cancer and non-cancer cells and are closely involved in the occurrence and development of CRC (43, 44). Moreover, studies have shown that exosomes are much easier to take up by cancerous cells than other vesicles of an equal amount, indicating that exosomes have a higher selectivity for cancer targeting (45). We particularly focused on the roles of miRNAs, lncRNAs, and circRNAs in the TME of CRC (**Table 1**).

**Table 1:** Roles of exosomal ncRNAs in the TME of CRC.

Exosomal ncRNAs	Source cell	Expression	Molecular axis	Functions	Ref.
miR-10b	CRC cells	Up	miR-10b/PI3K/Akt/mTOR	Promote CRC growth	(46)
miR-16-5p	BMSCs	Up	miR-16-5p/ ITGA2	Reduce CRC cell proliferation, migration and invasion	(47)
miR-221/222	CRC cells	Up	miR-221or222/ SPINT1/ HGF	Promote CRC cell invasiveness	(48)
miR-22-3p	BMSCs	Up	miR-22-3p/RAP2B/PI3K/ AKT	Inhibit CRC cell proliferation and invasion	(49)
miR-106b-3p	CRC cells	Up	miR-106b-3p/ DLC-1	Promote CRC cell invasiveness and metastasis	(50)
miR-17-5p	CAFs	Up	miR-17-5p /RUNX3 / MYC	Promote CRC cell aggressive phenotype	(51)
lncRNA MALAT1	CRC cells	Up	Lnc MALAT1/PI3K/Akt / mTOR/miR-26a/26b	Promote the CRC cell invasion and metastasis	(52)
LINC00659	CAFs	Up	LINC00659/miR-342-3p/ ANXA2	Promote CRC cell proliferation, invasion and migration	(53)
lncRNA UCA1	CAFs	Up	lncRNA UCA1/ceRNA	Promote CRC migration	(54)
lncRNA NNT-AS1	CRC cells	Up	lncRNA NNT-AS1/miR-496/RAP2C	Promote CRC cell proliferation, migration, and invasion	(55)
circ-ABCC1	CRC cells	Up	circ-ABCC1/Wnt/β-catenin	Promote cell stemness, sphere formation, and metastasis	(56)
circ_PTPRA	CRC cells	Down	circ_PTPRA/miR-671-5p-SMAD4	Inhibit CRC tumor growth	(57)
circCOG2	CRC cells	Up	circCOG2/miR-1305/TGF-β2/SMAD3	Promotes CRC cell proliferation, migration and invasion	(58)
circEPB41L2	CRC cells	Up	circEPB41L/miR-21-5p or miR-942-5p/PTEN/AKT	Inhibit CRC cell proliferation and migration	(59)

#### 2.2.1 miRNAs

Since the discovery that exosomes carry genetic material inside, scientists have conducted extensive research on exosomes. Currently, the role of exosomal miRNAs in cancer is the most studied. miRNA is an endogenous short ncRNA sequence that binds to the 3′ untranslated region (UTR) of a target mRNA to suppress its production by degrading or repressing translation (60). As we know, miRNAs are capable of regulating different cellular processes (61). However, when some of these mechanisms are changed and disrupted miRNA expression, tumor growth deviates from its typical course of progression (61). Increasing evidence shows that miRNA-carrying exosomes released from immune cells, mesenchymal cells, and cancer cells in the TME can shuttle from donor cells to recipient cells, even being taken up by distant cells to alter gene expression (62, 63).

Exosomal miR-19a is enriched in the serum of CRC patients as well as associated with poor prognosis (64). Treating CRC-bearing mice with tumor-derived exosomal miR-34a significantly reduced tumor size and prolonged survival of CRC-bearing mice (65). Dai et al. found CRC cell-derived exosomal miR-10b transferred to fibroblast cells and directly inhibited PIK3CA expression, reduced PI3K/Akt/mTOR pathway activity, and boosted TGF-β and SM α-actin expression (46). Exosomal miR-16-5p from bone marrow-derived mesenchymal stem cells (BMSCs) acted on CRC cells, reduced CRC cell proliferation, migration and invasion by reducing ITGA2 (47). According to Tian et al., CRC-derived exosomal miR-221/222 transferred to hepatic stromal cells decreased serine protease inhibitor Kunitz type 1(SPINT1) expression to activate liver hepatocyte growth factor (HGF), which plays a vital role in the formation of pre-metastatic niche (PMN) effect, leading to CRC invasiveness (48). Moreover, CRC cell proliferation and invasion were inhibited by exosomal miR-22-3p from BMSCs, which inhibited the PI3K/AKT pathway by reducing RAP2B expression (49). CRC-derived exosomal miR-106b-3p promoted CRC cell invasiveness by targeting deleted in liver cancer-1(DLC-1) (50). CAFs-derived exosomal miR-17-5p targeted RUNX3 to increase TGF-β1 expression and activate the TGF-β signaling pathway. Furthermore, TGF-β1 in the TME activated CAFs, which in turn released additional exosomal miR-17-5p to CRC cells and resulted in a cancer-promoting feedback loop, finally promoting CRC aggressive phenotype (51). These findings suggested exosomal miRNAs expression is closely related to CRC progression.

#### 2.2.2 lncRNAs

lncRNAs have the characteristics of low expression, moderate sequence conservation, and high tissue-specific (66). What makes lncRNAs unique is that most of them are specifically expressed in certain conditions and tissues instead of having widespread roles (66, 67). Interestingly, lncRNAs can be preferentially sorted into exosomes, which are intercellular communication mediators and involved in CRC development (68).

According to great research findings, lncRNAs spread to cells *via* exosomes, shaping a favorable microenvironment for tumor cell growth (69). Compared with normal cell-derived exosomes, cancer cell-derived exosomes are enriched with specific lncRNAs, which further accelerate the malignant progression of cancer in recipient cells (61). Exosomal lncRNA MALAT1 secreted by metastatic CRC cells increased FUT4 expression and activated PI3K/Akt/mTOR to sponge miR-26a/26b in primary CRC cells, leading to CRC progression (52). CAFs-derived exosomes delivered LINC00659 to CRC cells and sponged miR-342-3p, which regulated ANXA2 for CRC cell proliferation, invasion and migration (53). Moreover, downregulation of lncRNA UCA1 in serum exosomes affected cell migration in CRC progression by controlling the ceRNA network (54). Through the miR-496/RAP2C axis, exosomal lncRNA NNT-AS1 promoted CRC cell proliferation, migration, and invasion (55). In addition, exosomal lnc CCAL and exosomal lnc CRNDE-h have been implicated in CRC progression (70, 71).

#### 2.2.3 circRNAs

Circular RNA (circRNA) is an endogenous ncRNA created by exon back-splicing (72). One study calculated the ratio of back splicing to forward splicing product reads between cellular and cell-derived exosomes, the data obtained showed that the ratio of circRNA levels to linear RNA levels was approximately 6-fold higher in exosomes than in cells. This finding indicated that circRNAs are more present in exosomes than linear RNA (73). Duo et al. also came to the above conclusion by identifying KRAS mutant (DKO-1), mutant/wild-type (DLD-1) and wild-type (DKs-8) circRNA expression profiles in cells and exosomes (74). Moreover, numerous studies have shown that circRNAs are abundant and stable in exosomes and can translocate to nearby or distant cells and performed their functions (75, 76). Zhao et al. found that exosomal circ-ABCC1 from CRC cells promoted cell stemness, sphere formation, and metastasis by activating the Wnt/β-catenin pathway (56). Yang et al. found that exosomal circ_PTPRA inhibited CRC tumor growth by modulating the miR-671-5p/SMAD4 network (57). Through the miR-1305/TGF-β2/SMAD3 pathway, exosomal circCOG2 can transmit from cancer cells with high metastatic potential to cancer cells with low metastatic potential, promoting CRC proliferation, migration and invasion (58). Additionally, CRC cell-derived exosomal circEPB41L2 sponged miR-21-5p and miR-942-5p to inhibit proliferation and migration of CRC cells by regulating the PTEN/AKT signaling pathway (59).

## 3 Possible Functions and Mechanisms of Exosome-Derived ncRNAs in TME of CRC

**Figure 1:**
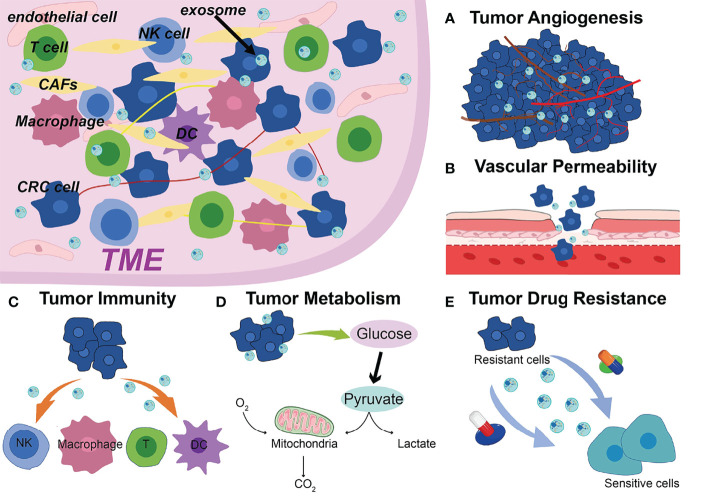
Exosomal ncRNAs contribute to the **(A)** tumor angiogenesis, **(B)** vascular permeability, **(C)** tumor immunity, **(D)** tumor metabolism, **(E)** drug resistance in the TME of CRC.

**Figure 2:**
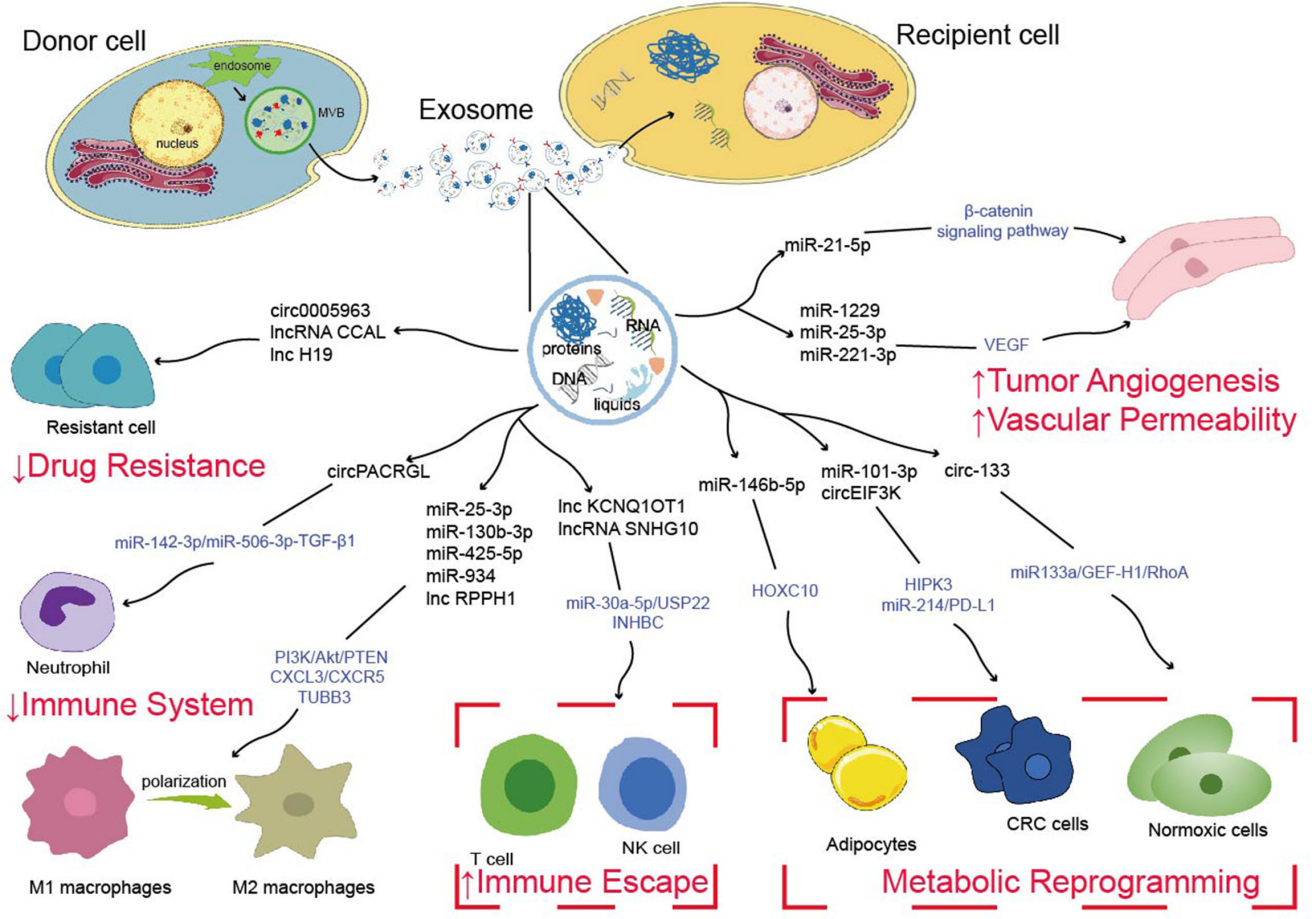
Possible mechanisms of exosome-derived ncRNAs to regulate tumor metastasis, tumor immunity, tumor metabolism and drug resistance.

The occurrence and progression of CRC require various complicated procedures (77). Metastasis is frequently the most dangerous and primary cause of CRC treatment failure which goes through a complex chain of events (78, 79). Abundant studies have shown that ncRNAs are significantly enriched in exosomes, which spread to the TME and remodel TME, ultimately leading to tumor metastasis, such as angiogenesis, vascular permeability, tumor immunity, tumor metabolism, drug resistance (**Figure 1**) (80–82). Additionally, drug resistance is closely linked to TME, exosomal ncRNAs play key roles in tumor cell adaptation to the TME and drug resistance (83). Based on the current studies, we discussed the role and mechanism of exosomal ncRNAs in TME of CRC in this section (**Figure 2**).

### 3.1 Exosome-Derived ncRNAs Promote Colorectal Tumor Angiogenesis and Vascular Permeability

One of the distinguishing characteristics of cancer is its ability to stimulate angiogenesis (84). In practice, TME needs to provide more oxygen and nutrients as the tumor continues to grow (23). During this process, TME sends signals to endothelial cells, causing an increase in the expression of mutiple angiogenic factors in order to form a new blood vessel (85). In particular, vascular endothelial growth factor (VEGF) is a powerful angiogenic factor that is widely recognized as a critical element in angiogenesis (86). VEGF regulates vessel formation by binding to VEGF receptors (VEGFRs)-1, -2, and -3, which are expressed on vascular endothelial cells (87). To create innovative anti-angiogenic medicines, it will be necessary to have a deeper knowledge of the cellular and molecular pathways that are involved in tumor angiogenesis.

Abnormal expression of exosomal ncRNAs impact cancer progression through regulating angiogenesis (88). He et al. found that miR-21-5p is propagated from CRC cells to recipient human umbilical vein endothelial cells (HUVECs) *via* exosomes, then activated β-catenin signaling pathway, increased downstream target expression, and regulated CRC angiogenesis and vascular permeability (89). In another study suggested significant upregulation of CRC cell-derived exosomal miR-1229 suppressed HIPK protein expression and activated the VEGF pathway of HUVECs. Moreover, exosomal miR-1229 inhibitor substantially inhibited tumor growth and angiogenesis as demonstrated by a nude mouse xenograft model (90). Zeng et al. showed that cancer-derived exosomal miR-25-3p drives CRC development by altering the expression of VEGFR2, ZO-1, occludin and Claudin5 in endothelial cells through targeting KLF2 and KLF4 (91). Likewise, the STAT3/VEGFR-2 signaling axis was activated by CRC cell-derived exosomal miR-221-3p, which promoted endothelial cells angiogenesis (92). Shang et al. discovered that miR-185-3p was significantly expressed in cancer cell-derived exosomes and overexpression of its target gene FOXO1 could reverse the increase in angiogenesis-related proteins caused by miR-183-5p in HMEC-1 cells (93). Chen et al. revealed that cancer cells derived exosomal miR-27b-3p trafficking into vascular endothelial cells reduced VE-Cad and p120 expression, increased vascular permeability *in vivo*, and eventually accelerated CRC metastasis (94). These studies demonstrate that exosomal ncRNAs affect angiogenesis and permeability to promote colorectal tumor metastasis.

### 3.2 Exosome-Derived ncRNAs Regulate Colorectal Tumor Immunity

Tumor-associated immune cells are a part of the TME, which play an important role in the TME and may have tumor-promoting or opposing effects (95, 96). Dunn et al. proposed the “cancer immunoediting” theory, which is based on the dual role of immunity in the complex interaction between tumor and host (97). In short, the immune system has the ability to prevent tumor development and progression while suppressing its occurrence and development (96, 97). However, some initial tumors can evade this attack and continue to develop in the host, increasing their chances of metastasis and recurrence (98). Interactions between immune cells and cancer cells, as well as exosome-related intercellular communication, are vital in tumor immune regulation, producing an immunosuppressive environment that promotes cancer development and progression (99).

Multiple studies demonstrate that exosomal ncRNAs mediate complex interactions between tumor and immune cells and induce changes in the expression of genes that regulate immunosuppression, confirming the functional significance of exosomal ncRNAs in immune modulation (100, 101). Another study showed that exosomal circPACRGL generated from CRC cells promoted neutrophil N1-N2 differentiation through the miR-142-3p/miR-506-3p-TGF-β1 axis (102). Moreover, the immune system normally responds to foreign antigens by promoting the proliferation and differentiation of cytotoxic T cells in TME (103). Xian et al. found that overexpression of exosomal lncRNA KCNQ1OT1 secreted by CRC cell affected cytotoxic T cells by regulating PD-L1 ubiquitination *via* miR-30a-5p/USP22, leading to immune escape (104). Similarly, exosomes ncRNAs can potentially serve as a bridge between tumor cells and NK cells, facilitating the exchange of information. For instance, exosomal lncRNA SNHG10 derived from CRC cells contributed to immune escape by suppressing NK cell function by upregulating INHBC expression as well (105).

Macrophages are innate immune cells that play a variety of roles in host defense and tissue homeostasis in TME (106). Exosomes have also been found to affect the pre-metastatic niche and promote cancer metastasis by altering the localization and function of tumor-associated macrophages (TAMs) through related mechanisms (11, 107, 108). Wang et al. found that when CXCL12/CXCR4 axis is activated, CRC cells produce exosomal miRNAs (miR-25-3p, miR-130b-3p, and miR-425-5p), which macrophages may take up and ​target PTEN through activation of PI3K/Akt signaling pathway, resulting in the M2 phenotypic transfer. Crucially, M2-polarized macrophages in turn secrete VEGF, which promotes CRC angiogenesis and liver metastases (109). Zhao et al. showed that tumor-derived exosomal miR-934-induced M2 macrophage polarization promotes CRC liver metastasis through activation of the CXCL13/CXCR5 axis (110). Meanwhile, another study found that CRC cell-derived exosomal lnc-RPPH1 promotes CRC cell metastasis and proliferation *in vivo* through mediating macrophage M2 polarization by binding to TUBB3 (111).

These studies confirmed that exosomal ncRNAs can alter the immunological microenvironment *via* influencing immune cell phenotypic.

### 3.3 Exosome-Derived ncRNAs Reprogram Colorectal Tumor Metabolism

In addition, reprogramming of energy metabolism is also seen as a hallmark of cancer (84). Glycolysis is the primary source of energy metabolism in tumors and obtains more sugar breakdown capacity, which can convert glucose into lactate to generate ATP, resulting in the formation of an acidic TME that is more conducive to cancer growth (112). Tao et al. found that exosomal miR-101-3p targeted and decreased HIPK3 expression in CRC cells, reduced mitochondrial membrane potential and produced reactive oxygen species (ROS), while increasing aerobic glycolysis and encouraging colorectal tumor development (113). Exosomal circ_0005963 from drug-resistant cells were delivered to drug-sensitive cells to inhibit glycolysis *via* the circ_0005963/miR-122/PKM2 pathway (114). Moreover, exosomes can mediate communication between cancer cells and adipocytes, which facilitate the transfer of nutrients such as lipids in the TME (115). Exosomal miR-146b-5p was released by cancer cells to promote browning of white adipose tissue (WATs) and increase lipolysis. Further study indicated that exosomal miR-146b-5p inhibited HOXC10 overexpression to increase WAT browning, reduce oxygen consumption and regulate lipolysis (116).

Furthermore, the nutritional status of oxygen has a substantial impact on cancer’s ability to use energy, and differences in energy storage between normoxic and hypoxic cells present various transfer potentials in the TME (117). Exosomal circ-133 generated from Hypoxic cells was transported into normoxic cells and promoted CRC metastasis by acting on the miR-133a/GEF-H1/RhoA axis (117). Yang et al. found that CAFs-derived exosomal circEIF3K was delivered to CRC cells and promoted CRC progression by regulating the miR-214/PD-L1 axis in the hypoxic microenvironment (118). According to these studies, targeting exosome cargoes that govern energy metabolism might provide a novel and successful method for cancer therapy.

### 3.4 Exosome-Derived ncRNAs Impact Colorectal Tumor Drug Resistance

Exosomal ncRNAs are involved not only in cancer progression and metastasis, but also in treatment resistance development (119). Obviously, one of the challenges in the treatment of tumor processes during chemoradiotherapy is the development of drug resistance. Exosomes present important mediators of intercellular communication, may contribute to the horizontal spread of drug resistance in heterogeneous cancer cell populations, which might make it impossible to treat many cancers effectively (120).

Methotrexate (MTX) is an antineoplastic drug that is widely used as standard chemotherapy in the treatment of various malignancies (121). Under MTX treatment, downregulation of the CDX2/HEPH axis by CAFs-derived exosomal miR-24-3p inhibitor accelerates the resistance of colon cancer cells to MTX (122). Recently, studies have demonstrated that exosomal miR-208b promotes regulatory T cells (Tregs) expansion by targeting PDCD4, and exosomal miR-208b upregulation was the most pronounced in oxaliplatin-resistant cells. That may be associated with reduced oxaliplatin-based chemosensitivity in CRC (123). Exosomal miR-208b can thus be employed as an oxaliplatin treatment response indicator. CAFs delivered exosomal lncRNA CCAL to cancer cells inhibited apoptosis in CRC cells, activated the β-catenin pathway, and promoted oxaliplatin resistance both *in vitro* and *in vivo* (70). Hon et al. found that exosomal circ-0000338 can transfer chemoresistance from FOLFOX-resistant cells to sensitive cells (124).

Furthermore, CRC patients who are undergoing chemotherapy commonly develop resistance to the drug 5-fluorouracil (5-FU) (125, 126). Similarly, overexpression of exosomal miR-181d-5p suppressed 5-FU sensitivity of CRC cells (127). According to Hu et al., exosomal miR-92a-3p was shown to be considerably higher in 5-FU/L-OHP resistant CRC patients than in 5-FU/L-OHP sensitive CRC patients. Further research found that CAFs-derived exosomal miR-92a-3p transfer to CRC cells then promoted the Wnt/β-catenin pathway, directly inhibited FBXW7 and MOAP1 to suppress mitochondrial apoptosis, which affects cancer growth and medication resistance (128). In addition, Ren et al. discovered that exosomal lncRNA H19 acted as a competitive endogenous RNA sponge for miR-141 to activate the Wnt/β-linked protein pathway, hence increasing resistance to oxaliplatin in CRC cells (129). These results implied that exosomal miRNAs might be as useful biomarkers for the development of CRC metastases and chemoresistance.

The above-mentioned evidence revealed that using exosomal ncRNAs to induce tumor cell resistance to anticancer drugs, cancer-associated cells were reprogrammed in the TME. We can determine that exosomal ncRNAs can also be potential therapeutic targets for overcoming drug resistance, thereby contributing to cancer patients.

## 4 Treatment Strategies for CRC Targeting ncRNA Derived from Exosomes

### 4.1 Exosome-Derived ncRNA as a Potential Colorectal Tumor Biomarker

CRC can be a preventable and treatable disease, and with current treatment, early diagnosis can greatly improve five-year survival rates (130). Biomarkers in bodily fluids can help identify the presence of cancer, metastasize and assess the response of treatment. Apparently, the methods of obtaining bodily fluids are significantly less invasive than traditional biopsies (131). Since exosomes can be detected in all bodily fluids and are produced by all cells, exosomes are great liquid biopsies that can be used to track the progression of sickness over time (37). Moreover, exosomal ncRNA is stable, abundant, reproducible and disease-specific, which lays the foundation for the early diagnosis of CRC (132, 133).

Min et al., found that miRNAs in exosomes, such as Let-7b-3p, miR-139-3p, miR-145-3p and miR150-3p, can be used as biomarkers for early diagnosis of CRC and have been validated in large cohorts (134). Besides, they had more discriminative power for multiple miRNA-binding diagnoses than for single miRNA-binding diagnoses (134). Moreover, high level of exosomal miR-19a expression in serum suggest the possibility of CRC recurrence (64). Pan et al. screened circ-0004771 as a potential diagnostic biomarker for CRC, and the ROC curve area of circ0004771 can distinguish benign intestinal diseases (BID), stage I/II CRC patients, and healthy controls (HCs) (135). Zhao et al. studied the exosomes taken from the plasma of CRC patients and discovered that the more severe the CRC patients, the lower the expression of miR-193a and the higher the expression of let-7g (136). Exosomal circR1 had specificity in CRC diagnosis and was associated with overall survival. In addition, the combination of exosomal circLPAR1, CEA, and CA19-9 enhanced the AUC value to 0.875 (137). As reported by Gao et al., CRC patients with high serum lncRNA 91H expression levels were generally at a higher risk of tumor recurrence or metastasis than other patients. Furthermore, CRC recurrence or metastasis can be predicted by detecting serum exosomal lncRNA 91H (138).

The above examples all demonstrate the potential and possibility of exosomal ncRNAs in various aspects, such as differential diagnosis, prognostic diagnosis, and staging characteristics.

### 4.2 Potential Application of Exosome-Derived ncRNAs in CRC Therapy

Numerous studies have established that exosomes serve crucial biological functions in cancer growth. Therefore, specifically targeting exosomes may be a promising therapeutic approach. Interfering with CRC progression by modulating exosome expression or blocking its transport pathway may be a therapeutic strategy.

Exosomes, with their lipid bilayer structure, can delivery anti-tumor chemicals to target cells, so as to achieve the effect of treatment (139–141). Researchers have packaged interfering RNAs and chemical drugs into exosomes through various methods and targeted transport to specific cells (142, 143). This engineered exosome technology can achieve the purpose of treating disease areas or cells. Tian et al. targeted exosomes by intravenous injection to specifically deliver doxorubicin (DOX) to tumor tissue in nude mice to inhibit tumor growth (144). Bagheri et al. created a MUC1 aptamer-modified exosome for DOX administration and showed promising efficacy in reducing colorectal tumor development *in vivo*, laying the groundwork for the use of exosomes in preclinical cancer treatment (145). Asadirad et al. found that exosomes carrying miR-155 could stimulate bone marrow-derived DC cells to release inflammatory factors IL-12p70 and IFN-γto achieve anti-CRC effect (146). Furthermore, Zhan et al. designed exosomes as a nanoplatform capable of precisely delivering drugs and miR-21i to tumor cells, and animals treated with this technique showed increased tumor suppression without severe adverse effects (147). Liang et al. used exosome engineering to deliver the combination of miR-21 and the chemotherapeutic drug 5-FU to recipient cells, which effectively overcame drug resistance and increased the cytotoxicity of 5-FU-resistant colon cancer cells (148). With the continuous refinement and diversification of engineered exosome technology, the exosomal drug delivery system holds a lot of promise for use in the medical field as a natural nanoscale drug delivery platform.

Taken together, the potential applications of exosome-derived ncRNAs in cancer therapy are not limited to these aspects. Their roles in TME have great potential and application prospects in the future.

## Conclusion

Exosome-derived ncRNAs play non-negligible roles in reshaping the CRC microenvironment. They serve as novel means of cell-to-cell crosstalk in TME, regulating signaling pathways that occupy their roles in cancer development and progression. Although research progress on functions and mechanisms of exosomal ncRNAs provide broad prospects for cancer diagnosis and therapeutic applications, the gap between their discovery and clinical practice cannot be ignored.

In the continuous research on the clinical application of exosomes, the technology of sensitive and accurate separation and detection of exosomes has not yet reached the expected level. Additionally, given the various advantages of exosomes, further research on how to maximize their loading efficiency is also required. The application of exosomes to cancer therapy will undoubtedly accelerate as more undiscovered areas are explored.

## Data Availability Statement

The original contributions presented in the study are included in the article/supplementary material. Further inquiries can be directed to the corresponding author.

## Author Contributions

XC and YZ were involved in the conception of the study. XC and MJ were involved in writing the article. JJ, ZZ and YZ critically revised the manuscript. All authors have read and approved the final manuscript.

## Funding

This study was supported by The National Natural Science Foundation of China (grant no. 8217121619 to YZ).

## Conflict of Interest

The authors declare that the research was conducted in the absence of any commercial or financial relationships that could be construed as a potential conflict of interest.

## Publisher’s Note

All claims expressed in this article are solely those of the authors and do not necessarily represent those of their affiliated organizations, or those of the publisher, the editors and the reviewers. Any product that may be evaluated in this article, or claim that may be made by its manufacturer, is not guaranteed or endorsed by the publisher.
